# Editorial: Movement disorders in neurometabolic conditions

**DOI:** 10.3389/fneur.2024.1397998

**Published:** 2024-03-22

**Authors:** Divyani Garg, Suvasini Sharma, Shekeeb S. Mohammad, Asuri Narayan Prasad

**Affiliations:** ^1^Department of Neurology, All India Institute of Medical Sciences, New Delhi, India; ^2^Department of Pediatrics (Neurology Division), Lady Hardinge Medical College and Associated Hospitals, New Delhi, India; ^3^Children's Hospital at Westmead, University of Sydney, Sydney, NSW, Australia; ^4^Schulich School of Medicine and Dentistry, London, ON, Canada

**Keywords:** movement disorders, metabolic disease, mitochondrial, DBS (deep brain stimulation), neurodegeneration with brain iron accumulation (NBIA)

Neurometabolic disorders (NMD) are a heterogeneous group of neurological conditions, arising from genetic disorders that involve metabolic pathways. These predominantly affect the nervous system, although they are usually systemic disorders (1). Clinical phenotypes tend to be complex, with admixture of neurobehavioral syndromes, developmental regression, ataxia, epilepsy, pyramidal and extrapyramidal involvement, and hypotonia. The common NMDs associated with movement disorders include metal storage disorders, neurotransmitter defects, lysosomal storage abnormalities, and disordered energy metabolism pathways. The movement disorders phenotype may offer diagnostic clues to the underlying NMD. Several NMDs are being considered as potentially treatable conditions, and their awareness is thus imperative amongst movement disorders specialists, neurologists, and pediatricians. Through the collection of articles in this Research Topic, we highlight the genotypic and phenotypic diversity of NMD and review current treatments including surgical management. The articles published under the aegis of this Research Topic represent an interesting amalgamation of these aspects.

Despite the increasing uptake of deep brain stimulation (DBS) for childhood onset movement disorders, the evidence for the role of DBS in rare NMDs is still based on few reports. Three manuscripts in this issue further add to the understanding of DBS and possible alternative brain targets such as the sub-thalamic nucleus (STN) or the pediculopontine nucleus (PPN) in addition to the classic Globus pallidus interna (Gpi) targeting for dystonia. Vera and Gropman, report beneficial effects for dystonia in patients with atypical compared to typical Pantothenate Kinase Associated Neurodegeneration (PKAN), a Neurodegeneration with Brain Iron Accumulation (NBIA) arising from variants in the *PANK2* gene. Despite the progressive loss of benefit seen early with DBS, there was still better function in the Burke-Fahn-Marsden Dystonia rating scale, motor (BFMDRS-M) at 5 years. Another NMD amenable to DBS noted in this review was Lesch Nyhan disease, wherein Gpi-DBS was associated with greater improvement in self-injurious behavior compared to dystonia. Expanding the clinical application of DBS in NMDs, Nataraj et al. report the use of DBS with personalized targeting approach in two siblings with Mitochondrial Enoyl CoA Reductase (MEPAN) syndrome. MEPAN syndrome is an ultra-rare pediatric NMD arising from autosomal recessive loss-of-function mutations in the *MECR* gene, and it affects mitochondrial fatty acid synthesis (2). Affected children present in childhood with severe dystonia, optic atrophy, and basal ganglia abnormalities. In this report, the surgical process had three stages, and benefit in dystonia was noted up to 1 year after the procedure. This report provides evidence for benefit of DBS amongst children with this rare NMD as well as insights into dual targeting in DBS.

The Research Topic features several reports of rare NMDS with treatment responses in some. An interesting case report describes a late-onset Molybdenum cofactor deficiency (MoCD) type A presenting as a Leigh-like phenotype (Almudhry, Prasad, Rupar, Tay, Ratko et al.). MoCD may present as an early- or late-onset neonatal encephalopathy (3). Depending on the impairment, MoCD may be classified as types A, B, or C. Type A may be reversible by administration of cyclic pyranopterin (fosdenopterin) (4). In this report, the patient manifested at age 5 months, with dyskinesias, developmental regression, and hypotonia associated with a febrile illness. MRI showed a Leigh-like picture, with asymmetrical globus pallidi signal change with lactate accumulation on MRS suggesting an impairment in energy metabolism. However, genomic sequencing helped to resolve the genetic basis of the disorder.

In a case series of five patients, clinical features of children with the mitochondrial DNA depletion syndrome (MDDS) (Almudhry, Saini et al.) are delineated. MDDS are clinically heterogeneous, and four groups of presentations are known: myopathic, heptocerebral, encephalomyopathic, and neurogastrointestinal (5). Mutations in *SUCLA2* and *SUCLG1* code for subunits of the enzyme succinyl coA synthase, and variants usually lead to an encephalomyopathic presentation. In this series, children presented with variable deficits, hyperkinetic movement disorders, neurodevelopmental regression, and hypotonia. Children with *SUCLG1*-related disorder displayed greater severity of symptoms at presentation. Biochemically, these were characterized by the presence of methylmalonic aciduria, elevated lactate, C3, C4DC, and C5-PH acylcarnitine.

Copper deficiency is associated with several neurological syndromes, which comprise myelopathy, optic neuropathy, and demyelination. In an intriguing case report, Benkirane et al. describe a patient who initially presented with clinical and radiological features of myelopathy, low serum copper, and ceruloplasmin levels. Despite copper supplementation, copper-related parameters remained abnormal and progressive deterioration with onset of amyotrophy was observed. Genetic testing for genes related to both copper transport and ALS were not found. The report offers insights into the interface between copper transport and pathogenic processes underlying ALS.

PEBEL1 (progressive encephalopathy with brain edema and/or leukoencephalopathy) is a rare NMD due to mutations in the *NAXE* gene. *NAXE* encodes the NAD(P)HX epimerase, required for restoration of NADH and NADPH (6). It is a severe encephalopathy, with rapid deterioration and death in association with intercurrent infections in childhood. Almudhry, Prasad, Rupar, Tay, Prasad report a patient with PEBEL1 presenting a milder phenotype (neurological deterioration was seen in association with febrile infections) with persistent residual deficits. A remarkable and notable stabilization followed institution of a mitochondrial cocktail in which niacin could be a component most likely to have led to improvement.

In conclusion, NMDs are rare conditions that can present and mimic more common movement disorders. Their recognition can be rewarding particularly if interventions can be made to modify clinical course and natural history of the underlying condition. DBS should not be discarded as a therapeutic option in NMDs, particularly in cases with refractory dystonia and alternative brain targets may be considered. This issue highlights some novel clinical, biochemical, and treatment aspects of rare NMDs, which arguably will be a group of disorders that will be more amenable to refining precision therapies as their biochemical basis is better understood compared to many novel rare monogenic disorders.

## Author contributions

DG: Writing – review & editing, Writing – original draft, Data curation, Conceptualization. SS: Writing – review & editing, Writing – original draft, Supervision, Conceptualization. SM: Writing – review & editing, Writing – original draft, Validation, Supervision, Conceptualization. AP: Writing – review & editing, Writing – original draft, Validation, Supervision, Conceptualization.
